# A clinical nomogram incorporating hyperhomocysteinemia for predicting severe delayed encephalopathy after acute carbon monoxide poisoning: a retrospective single-center study

**DOI:** 10.3389/fmed.2026.1857056

**Published:** 2026-06-24

**Authors:** Wanqiu Zhu, Yi Zhang, Fang Liang, Shuhua Yuan, Yu Gao, Xuehua Liu, Xiaomin Hou, Linlin Ma, Jing Zhang, Ding Nan, Lu Yang, Yafei He, Jing Yang

**Affiliations:** Department of Hyperbaric Oxygen, Beijing Chao-Yang Hospital (Changying Campus), Capital Medical University, Beijing, China

**Keywords:** carbon monoxide poisoning, delayed encephalopathy, hyperhomocysteinemia, hypertension, nomogram, predictive model

## Abstract

**Background:**

Severe delayed encephalopathy after acute carbon monoxide poisoning (s-DEACMP) is a disabling complication associated with substantial long-term neurological impairment, yet reliable predictors for risk stratification during acute hospitalization remain limited. This study aimed to identify predictors associated with s-DEACMP and to develop a preliminary predictive model.

**Methods:**

In this retrospective single-center cohort study, 200 patients with acute carbon monoxide poisoning (ACMP) admitted between 2017 and 2024 were analyzed. Patients were categorized into DEACMP (*n* = 140) and non-DEACMP (*n* = 60) groups; the DEACMP group was further stratified into severe (s-DEACMP, *n* = 97) and mild-to-moderate (m-DEACMP, *n* = 43) subgroups based on Activities of Daily Living scores assessed at peak disease severity during hospitalization. Clinical characteristics were compared between the s-DEACMP group and a combined control group (n-DEACMP + m-DEACMP). Independent predictors were identified using multivariate logistic regression. A nomogram was constructed and internally validated using receiver operating characteristic (ROC) curves, calibration analysis, and decision curve analysis (DCA).

**Results:**

Patients with s-DEACMP were significantly older and had longer duration of disturbance of consciousness, higher prevalence of hypertension and hyperhomocysteinemia, elevated D-dimer levels, and lower serum albumin concentrations compared with controls (all *P* < 0.05). Multivariate analysis identified age >40 years (OR = 31.90, 95% CI = 3.93–259.14), disturbance of consciousness >24 h (OR = 3.06, 95% CI = 1.58–5.94), hypertension (OR = 1.99, 95% CI = 1.02–3.90), and hyperhomocysteinemia (OR = 2.57, 95% CI = 1.24–5.31) as factors independently associated with s-DEACMP. The nomogram demonstrated acceptable discrimination and calibration.

**Conclusion:**

The proposed nomogram may assist in identifying patients at increased risk for s-DEACMP during acute hospitalization. External multicenter validation is required before broader clinical application.

## Introduction

Acute carbon monoxide poisoning (ACMP) is a common toxicological emergency and remains one of the leading causes of poisoning-related mortality in clinical practice. It results from acute exposure to carbon monoxide (CO), which induces systemic hypoxic–ischemic injury owing to CO’s strong affinity for hemoglobin (1, 2). After an initial period of recovery from impaired consciousness, a subset of patients develop delayed encephalopathy after acute carbon monoxide poisoning (DEACMP) within 2–60 days. DEACMP is a severe demyelinating disorder characterized by diverse neuropsychological deficits, including cognitive impairment (dementia, delirium), extrapyramidal manifestations (parkinsonism), pyramidal tract injury, and focal cortical dysfunction. Reported incidence rates vary widely in China, ranging from 0.2 to 47.3% (3). Several hypotheses have been proposed to explain the pathogenesis of DEACMP, primarily involving inflammatory and immune-mediated mechanisms, neuronal apoptosis, and direct neurotoxicity. However, these mechanisms alone do not fully account for the complex clinical manifestations and heterogeneous disease progression observed in clinical practice (4). Patients with severe DEACMP (s-DEACMP) often experience persistent neurological disability, profound loss of independence in activities of daily living, and require long-term rehabilitation or supportive care, thereby imposing a substantial socioeconomic burden on families and healthcare systems.

Previous studies have mainly focused on identifying predictors for the occurrence of DEACMP and evaluating their prognostic significance (5). However, limited research has specifically addressed predictors associated with severe DEACMP (s-DEACMP). In addition, currently available prediction models, such as the COGAS score, have primarily focused on overall neurocognitive outcomes after ACMP rather than severe delayed encephalopathy specifically (6). Risk stratification of patients at increased risk for severe neurological disability during acute hospitalization therefore remains a clinical challenge. Therefore, the present study aimed to systematically evaluate the clinical characteristics and predictors associated with s-DEACMP and to develop a preliminary prediction model based on readily available clinical and laboratory parameters. The goal of this model was to assist in identifying patients at increased risk for severe DEACMP during acute hospitalization prior to delayed neurological deterioration.

## Materials and methods

### Study ethics

This study was approved by the Medical Ethics Committee of Beijing Chao-Yang Hospital (Changying Campus), Capital Medical University (Approval No. LGH-2025-Sci-141). All procedures conformed to the principles of the Declaration of Helsinki. The requirement for written informed consent was waived by the Ethics Committee due to the retrospective nature of the study and the use of anonymized clinical data.

### Study design and patient source

This was a retrospective, single-center cohort study. Consecutive patients with acute carbon monoxide poisoning (ACMP) admitted to the Department of Hyperbaric Oxygen, Beijing Chao-Yang Hospital, Capital Medical University, between January 1, 2017, and December 31, 2024, were screened for eligibility. A total of 224 patients with ACMP were initially screened. After exclusion of 24 patients due to incomplete clinical data, co-exposure to other toxic gases, or alternative causes of disturbance of consciousness, 200 patients met the inclusion criteria and were ultimately enrolled in the final analysis.

### Inclusion and exclusion criteria

Inclusion criteria: Patients were diagnosed according to the Diagnostic Criteria of Occupational Acute Carbon Monoxide Poisoning formulated by the Ministry of Health, which required: (1) documented exposure to carbon monoxide; (2) comprehensive clinical evaluation confirming systemic involvement and vital organ dysfunction; and (3) laboratory confirmation of elevated carboxyhemoglobin (COHb), excluding false-positive results (1–3).

Exclusion criteria: (1) incomplete or missing key clinical or laboratory data; (2) disturbance of consciousness attributable to comorbid conditions (e.g., hypoglycemia, cardiopulmonary arrest); or (3) co-exposure to other toxic gases.

### Patient grouping

**FIGURE 1:**
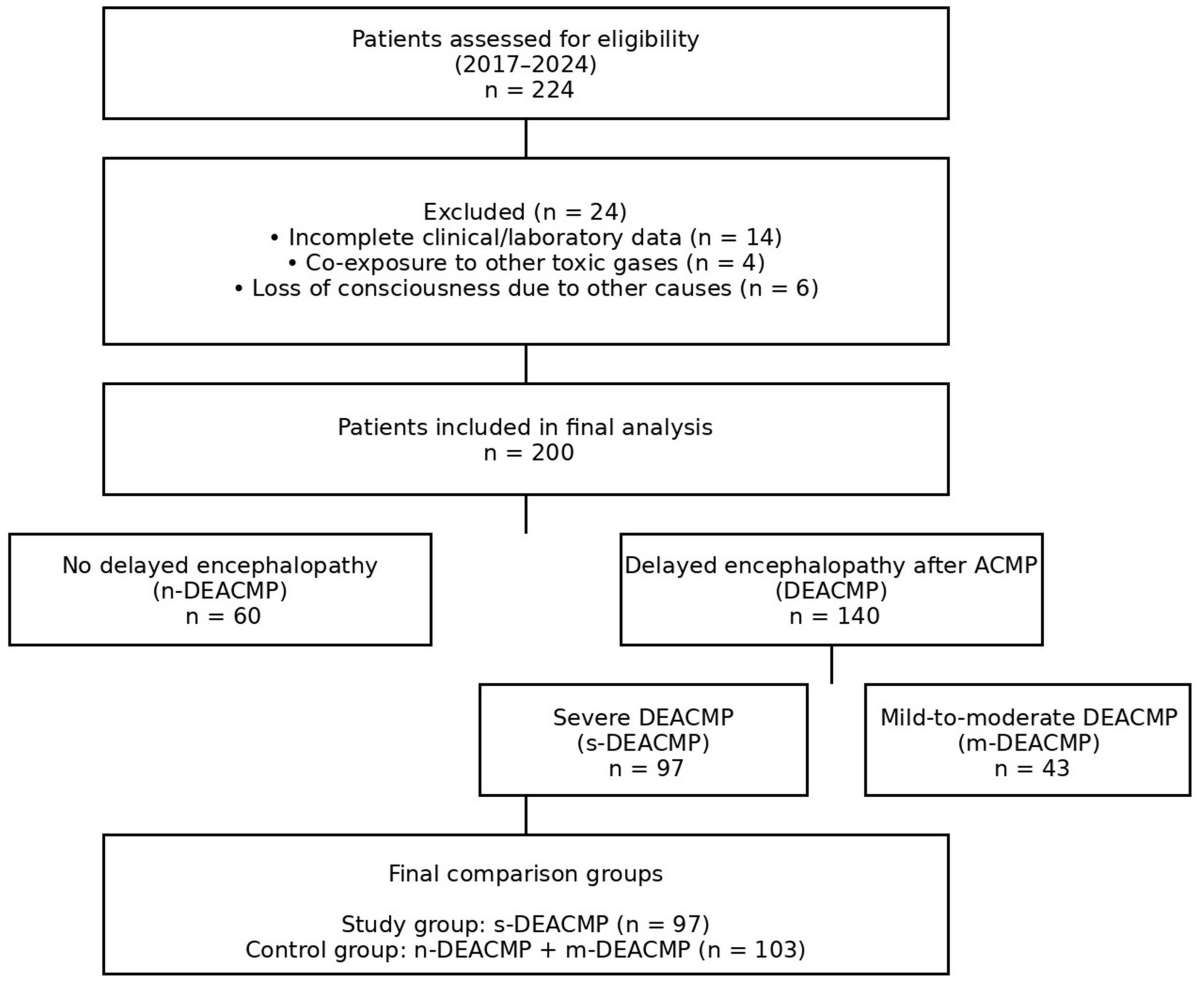
Flowchart of patient selection. A total of 224 patients with acute carbon monoxide poisoning (ACMP) were initially screened. After exclusion of 24 patients due to incomplete clinical data, co-exposure to other toxic gases, or alternative causes of disturbance of consciousness, 200 patients were included in the final analysis. These patients were classified into a control group (*n* = 103; non-DEACMP + mild-to-moderate DEACMP) and a study group (*n* = 97; severe DEACMP).

Patients were classified into delayed encephalopathy after acute carbon monoxide poisoning (DEACMP) and non-DEACMP (n-DEACMP) groups. Patients with DEACMP were further stratified according to Activities of Daily Living (ADL) scores at peak disease severity. ADL was assessed using the Barthel Index scale (range: 0–100), with lower scores indicating greater functional dependence. Assessments were performed by trained clinicians during hospitalization. Peak disease severity was defined as the period during hospitalization when neurological dysfunction and functional impairment were most pronounced based on comprehensive clinical evaluation. An ADL score ≤ 60 was defined as severe DEACMP (s-DEACMP), indicating dependence in daily activities, whereas scores > 60 indicated mild-to-moderate DEACMP (m-DEACMP), reflecting preserved independence. Clinical characteristics of the s-DEACMP group were compared with those of the combined control group (n-DEACMP + m-DEACMP**).** Accordingly, the prediction target of the present model was severe DEACMP versus a combined comparator group consisting of patients without DEACMP and those with mild-to-moderate DEACMP. The patient enrollment process is illustrated in Figure 1.

### Data collection

Data were extracted from medical records, including demographic and clinical characteristics, laboratory parameters obtained within 12 h of admission, neuroimaging findings, and clinical manifestations of ACMP and DEACMP. Patients with missing data were excluded from the analysis. Cases with incomplete key clinical or laboratory variables were excluded prior to statistical analysis, and no data imputation procedures were performed because the proportion of missing data was low. Laboratory parameters were interpreted according to standardized institutional reference ranges used in routine clinical practice. Collected variables included: (1) General information: sex, age, type of work (manual or mental labor), comorbidities, smoking history, and alcohol consumption; (2) Laboratory indicators within 12 h of admission: white blood cell count (WBC), hemoglobin (Hb), platelet count (PLT), neutrophil-to-lymphocyte ratio (NLR), platelet-to-lymphocyte ratio (PLR), monocyte-to-lymphocyte ratio (MLR), D-dimer, albumin (ALB), aspartate aminotransferase (AST), alanine aminotransferase (ALT), lactate dehydrogenase (LDH), total cholesterol (TC), high-density lipoprotein cholesterol (HDL-C), low-density lipoprotein cholesterol (LDL-C), homocysteine (HCY), and hemoglobin A1c (HbA1c); (3) Neuroimaging findings on brain magnetic resonance imaging (MRI); (4) Clinical features of ACMP and DEACMP, including the cause of poisoning, Glasgow Coma Scale (GCS) score on admission, duration of disturbance of consciousness, receipt of hyperbaric oxygen therapy, predisposing factors for DEACMP (e.g., infection, psychological stress), and the duration of the “pseudo-recovery period.” Hyperbaric oxygen therapy was administered according to institutional treatment protocols for ACMP; however, detailed information regarding treatment timing, pressure settings, and number of sessions was not consistently available for retrospective analysis. Comorbidities included hypertension, hyperlipidemia, diabetes mellitus, hyperhomocysteinemia, coronary heart disease, and cerebrovascular disease. Hyperhomocysteinemia was defined as a serum homocysteine concentration >15 μmol/L, measured either from laboratory testing performed within 12 h of admission or from documented prior medical records when available. In our institution, serum homocysteine testing is routinely available during acute hospitalization, and results are typically available within 24–48 h after sample collection. However, the availability and turnaround time of homocysteine testing may vary across institutions, which could influence the practical applicability of the nomogram in different clinical settings.

### Statistical analysis

Continuous variables were tested for normality and are presented as medians with interquartile ranges (IQRs) for non-normally distributed data. Categorical variables are expressed as numbers (percentages) and were compared using the chi-square test or Fisher’s exact test, as appropriate. Univariate logistic regression analysis was performed to identify potential predictors of s-DEACMP, followed by multivariate logistic regression to determine independent risk factors. Cutoff values for age ( > 40 years) and duration of disturbance of consciousness ( > 24 h) were selected based on previous literature and clinical interpretability. Results are reported as odds ratios (ORs) with 95% confidence intervals (CIs). All statistical analyses were conducted using SPSS version 27.0 (IBM Corp., Armonk, NY, United States), with statistical significance defined as *P* < 0.05. Patients were randomly divided into derivation and validation cohorts for model development and internal validation. Random allocation was performed using a computer-generated randomization procedure at an approximate ratio of 7:3. Model performance was evaluated using receiver operating characteristic (ROC) curves, calibration plots, and decision curve analysis (DCA). Given the relatively small validation cohort, the internal validation results should be interpreted cautiously.

## Results

### Baseline patient characteristics

Among the 200 patients with acute carbon monoxide poisoning (ACMP), 97 developed severe delayed encephalopathy (s-DEACMP), 43 developed mild-to-moderate DEACMP (m-DEACMP), and 60 did not develop delayed encephalopathy (n-DEACMP). In patients with s-DEACMP, the pseudo-recovery period ranged from 1 to 60 days; relapse was precipitated by infection in 12 cases and by psychological stress in 7 cases. Most cases of poisoning were associated with carbon monoxide exposure from heating-related sources. All patients with s-DEACMP presented with cognitive impairment, and a subset also exhibited extrapyramidal or psychiatric manifestations. Neuroimaging revealed characteristic brain lesions consistent with delayed encephalopathy. Compared with the control group (n-DEACMP + m-DEACMP), patients in the s-DEACMP group were significantly older (62 [55–66] vs. 53 [41–65] years, *P* < 0.001) and more frequently aged > 40 years (99.0% vs. 75.7%, *P* < 0.001). They were more often engaged in manual labor (87.6% vs. 66.0%, *P* < 0.001) and were more commonly exposed to CO from heating-related sources (94.8% vs. 78.6%, *P* < 0.001). Neurologically, the s-DEACMP group had lower Glasgow Coma Scale (GCS) scores on admission [median 3 (3–3) vs. 3 (3–7), *P* = 0.027], a longer duration of disturbance of consciousness [25 (8–48) vs. 12 (3–27) hours, *P* = 0.005], and a higher proportion of prolonged disturbance of consciousness > 24 h (50.0% vs. 26.0%, *P* < 0.001). In addition, patients with s-DEACMP showed a higher prevalence of hypertension (43.3% vs. 23.3%, *P* = 0.003) and hyperhomocysteinemia (37.1% vs. 24.3%, *P* = 0.049). Laboratory findings revealed significantly elevated D-dimer levels [0.86 (0.39–1.99) vs. 0.43 (0.25–1.23) mg/L, *P* = 0.003] and reduced serum albumin concentrations [38.7 (37.4–41.0) vs. 40.5 (38.4–42.7) g/L, *P* = 0.002] in the s-DEACMP group. Detailed baseline characteristics are presented in Table 1. Although hyperhomocysteinemia reached statistical significance, the borderline (*P* = 0.049) should be interpreted cautiously. Collectively, patients who developed s-DEACMP exhibited distinct demographic, neurological, and biochemical profiles, suggesting that advanced age, prolonged disturbance of consciousness, vascular comorbidities, and systemic metabolic disturbances may be associated with severe disease progression.

**TABLE 1:** Baseline characteristics of patients with s-DEACMP and the control group (n-DEACMP + m-DEACMP).

Variables	Control group (n-DEACMP + m-DEACMP) (*n* = 103)	Study group s-DEACMP (*n* = 97)	*P*-value
Sex, *n* (male/female)	62/41	48/49	0.128
Age, years, M (Q_1_, Q_3_)	53 (41, 65)	62 (55, 66)	<0.001*
Age > 40 years, *n* (%)	78 (75.7%)	96 (99.0%)	<0.001*
Type of work, *n* (manual/mental laborers)	68/35	85/12	<0.001*
Causes of CO poisoning			
Heating-related exposure, *n* (%)	81 (78.6%)	92 (94.8%)	<0.001*
Bathing and cooking, *n* (%)	17 (16.5%)	4 (4.1%)	0.004*
Suicide, *n* (%)	5 (4.9%)	1 (1.0%)	0.213
GCS score on admission, M (Q_1_, Q_3_)	3 (3, 7)	3 (3, 3)	0.027*
Duration of disturbance of consciousness, h, M (Q1, Q3)	12 (3, 27)	25 (8, 48)	0.005*
Disturbance of consciousness > 24 h, n/N (%)	26/100 (26.0%)	48/96 (50.0%)	<0.001*
Hyperbaric oxygen therapy for ACMP, n/N (%)	75/94 (79.8%)	72/97 (74.2%)	0.362
Underlying diseases			
Hypertension, *n* (%)	24 (23.3%)	42 (43.3%)	0.003*
Hyperlipidemia, *n* (%)	36 (35.0%)	32 (33.0%)	0.770
Diabetes mellitus, *n* (%)	22 (21.4%)	18 (18.6%)	0.620
Hyperhomocysteinemia, *n* (%)	25 (24.3%)	36 (37.1%)	0.049*
Coronary heart disease, *n* (%)	14 (13.6%)	8 (8.2%)	0.227
Cerebrovascular disease, *n* (%)	17 (16.5%)	21 (21.6%)	0.354
Smoking history, *n* (%)	30 (29.1%)	27 (27.8%)	0.840
Smoking index > 400 cigarette-years, *n* (%)	18 (17.5%)	20 (20.6%)	0.571
Alcohol history, *n* (%)	25 (24.3%)	23 (23.7%)	0.926
Laboratory indicators within 12 h of admission			
WBC, × 10^9^/L, M (Q_1_, Q_3_)	6.14 (5.00, 9.39)	6.43 (5.11, 7.66)	0.527
Hb, g/L, M (Q_1_, Q_3_)	133 (124, 146)	131 (123, 142)	0.150
PLT, × 10^9^/L, M (Q1, Q3)	216 (188, 258)	216 (179, 284)	0.836
NLR, M (Q_1_, Q_3_)	2.39 (1.71, 4.28)	2.66 (1.80, 3.73)	0.547
PLR, M (Q_1_, Q_3_)	140.52 (103.48, 190.57)	144.19 (107.89, 193.85)	0.523
MLR, M (Q_1_, Q_3_)	0.24 (0.18, 0.35)	0.23 (0.18, 0.29)	0.248
D-dimer, mg/L, M (Q_1_, Q_3_)	0.43 (0.25, 1.23)	0.86 (0.39, 1.99)	0.003*
ALB, g/L, M (Q_1_, Q_3_)	40.5 (38.4, 42.7)	38.7 (37.4, 41.0)	0.002*
AST, U/L, M (Q_1_, Q_3_)	23 (18, 40)	22 (18, 33)	0.668
ALT, U/L, M (Q_1_, Q_3_)	20 (14, 39)	21 (14, 31)	0.587
LDH, U/L, M (Q_1_, Q_3_)	182 (157, 246)	204 (171, 248)	0.145
TC, mmol/L, M (Q_1_, Q_3_)	4.47 (3.60, 5.36)	4.30 (3.58, 4.96)	0.215
HDL-C, mmol/L, M (Q_1_, Q_3_)	1.10 (0.90, 1.29)	1.04 (0.90, 1.30)	0.830
LDL-C, mmol/L, M (Q_1_, Q_3_)	2.63 (2.06, 3.37)	2.50 (2.00, 3.12)	0.195
TG, mmol/L, M (Q_1_, Q_3_)	1.44 (1.03, 2.04)	1.16 (0.90, 1.49)	0.102
HCY, μmol/L, M (Q_1_, Q_3_)	12 (10, 17)	14 (10, 16)	0.286
HbA1c, %, M (Q_1_, Q_3_)	5.8 (5.5, 6.2)	5.7 (5.4, 6.2)	0.440

M, median; Q_1_, first quartile; Q_3_, third quartile; GCS, Glasgow Coma Scale; ACMP, acute carbon monoxide poisoning; DEACMP, delayed encephalopathy after acute carbon monoxide poisoning; s-DEACMP, severe delayed encephalopathy after acute carbon monoxide poisoning; m-DEACMP, mild-to-moderate delayed encephalopathy after acute carbon monoxide poisoning; WBC, white blood cell count; Hb, hemoglobin; PLT, platelet count; NLR, neutrophil-to-lymphocyte ratio; PLR, platelet-to-lymphocyte ratio; MLR, monocyte-to-lymphocyte ratio; ALB, albumin; AST, aspartate aminotransferase; ALT, alanine aminotransferase; LDH, lactate dehydrogenase; TC, total cholesterol; HDL-C, high-density lipoprotein cholesterol; LDL-C, low-density lipoprotein cholesterol; TG, triglycerides; HCY, homocysteine; HbA1c, hemoglobin A1c; —, Fisher’s exact test. *P < 0.05. Some rows report n/N because of missing values (e.g., 26/100, 48/96, 75/94).

### Logistic regression and ROC analyses

Univariate logistic regression analysis identified ten variables significantly associated with the development of s-DEACMP, including age, age > 40 years, occupation (manual labor), GCS score on admission, GCS score ≤ 12, duration of disturbance of consciousness, disturbance of consciousness > 24 h, hypertension, hyperhomocysteinemia, and serum albumin levels (*P* ≤ 0.05; Table 2). To determine independent predictors, these variables were entered into a multivariate logistic regression model. Four factors remained independently associated with s-DEACMP: age > 40 years (OR = 31.90; 95% CI = 3.93–259.14; *P* = 0.001), disturbance of consciousness > 24 h (OR = 3.06; 95% CI = 1.58–5.94; *P* = 0.001), hypertension (OR = 1.99; 95% CI = 1.02–3.90; *P* = 0.045), and hyperhomocysteinemia (OR = 2.57; 95% CI = 1.24–5.31; *P* = 0.011) (Table 3). The large odds ratio and wide confidence interval observed for age > 40 years may reflect estimation instability related to the high proportion of older patients in the s-DEACMP group and should therefore be interpreted cautiously. Taken together, multivariate analysis demonstrated that advanced age, prolonged disturbance of consciousness, hypertension, and hyperhomocysteinemia were independently associated with severe delayed encephalopathy following acute carbon monoxide poisoning.

**TABLE 2:** Univariate logistic regression analysis for s-DEACMP.

Variables	*P-*value	OR	95% CI
Sex (female vs. male)	0.129	1.54	0.88–2.70
Age# (per year)	<0.001*	1.06	1.03–1.09
Age (>40 years vs. ≤ 40 years)	0.001*	30.77	4.08–232.18
Type of work (manual vs. mental laborers)	0.001*	3.65	1.76–7.56
GCS score on admission#	0.048*	0.92	0.85–1.00
GCS score ≤ 12 (yes vs. no)	0.050	2.89	1.00–8.37
Duration of disturbance of consciousness# (per hour)	0.017*	1.01	1.00–1.02
Disturbance of consciousness > 24 h (yes vs. no)	0.001*	2.85	1.56–5.19
Hyperbaric oxygen therapy for ACMP (no vs. yes)	0.362	0.73	0.37–1.44
Hypertension (yes vs. no)	0.003*	2.51	1.37–4.62
Hyperlipidemia (yes vs. no)	0.770	0.92	0.51–1.65
Diabetes mellitus (yes vs. no)	0.621	0.84	0.42–1.68
Hyperhomocysteinemia (yes vs. no)	0.048*	1.84	1.00–3.39
Coronary heart disease (yes vs. no)	0.232	0.57	0.23–1.43
Cerebrovascular disease (yes vs. no)	0.355	1.40	0.69–2.84
Smoking history (yes vs. no)	0.840	0.94	0.51–1.74
Smoking index > 400 cigarette-years (yes vs. no)	0.572	1.23	0.61–2.49
WBC#	0.147	0.94	0.87–1.02
Hb#	0.159	0.99	0.97–1.01
PLT#	0.500	1.00	1.00–1.01
NLR#	0.170	0.94	0.87–1.03
PLR#	0.800	1.00	1.00–1.01
MLR#	0.152	0.35	0.08–1.48
D-dimer#	0.336	1.05	0.95–1.16
ALB#	0.026*	0.92	0.85–0.99
AST#	0.301	1.00	1.00–1.01
ALT#	0.494	1.00	1.00–1.01
LDH#	0.416	1.00	0.99–1.00
TC#	0.134	0.81	0.62–1.07
HDL-C#	0.484	1.30	0.62–2.71
LDL-C#	0.089	0.76	0.55–1.04
TG#	0.094	0.80	0.61–1.04
HbA1c#	0.220	0.82	0.60–1.12

OR, odds ratio; CI, confidence interval; GCS, Glasgow Coma Scale; ACMP, acute carbon monoxide poisoning; WBC, white blood cell count; Hb, hemoglobin; PLT, platelet count; NLR, neutrophil-to-lymphocyte ratio; PLR, platelet-to-lymphocyte ratio; MLR, monocyte-to-lymphocyte ratio; ALB, albumin; AST, aspartate aminotransferase; ALT, alanine aminotransferase; LDH, lactate dehydrogenase; TC, total cholesterol; HCY, homocysteine; HDL-C, high-density lipoprotein cholesterol; LDL-C, low-density lipoprotein cholesterol; TG, triglycerides; HbA1c, hemoglobin A1c; #, continuous variable; *P ≤ 0.05.

**TABLE 3:** Multivariate logistic regression analysis for s-DEACMP.

Variables	*P*-value	OR	95% CI
Age ( > 40 years vs. ≤ 40 years)	0.001*	31.90	3.93–259.14
Disturbance of consciousness > 24 h (yes vs. no)	0.001*	3.06	1.58–5.94
Hypertension (yes vs. no)	0.045*	1.99	1.02–3.90
Hyperhomocysteinemia (yes vs. no)	0.011*	2.57	1.24–5.31
Constant	<0.001*	0.02	–

OR, odds ratio; CI, confidence interval. *P < 0.05.

### Construction and validation of the predictive model

**FIGURE 2:**
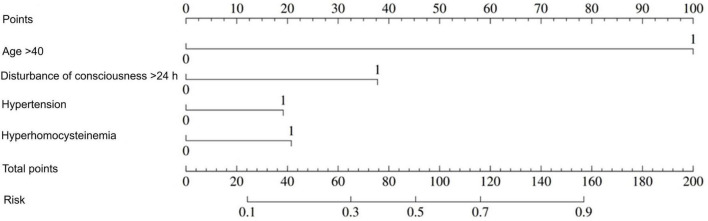
Nomogram for predicting severe delayed encephalopathy after acute carbon monoxide poisoning (s-DEACMP). The nomogram incorporates four independent predictors—age > 40 years, disturbance of consciousness > 24 h, hypertension, and hyperhomocysteinemia. Points assigned to each variable are summed to obtain a total score, which corresponds to the estimated probability of developing s-DEACMP.

**FIGURE 3:**
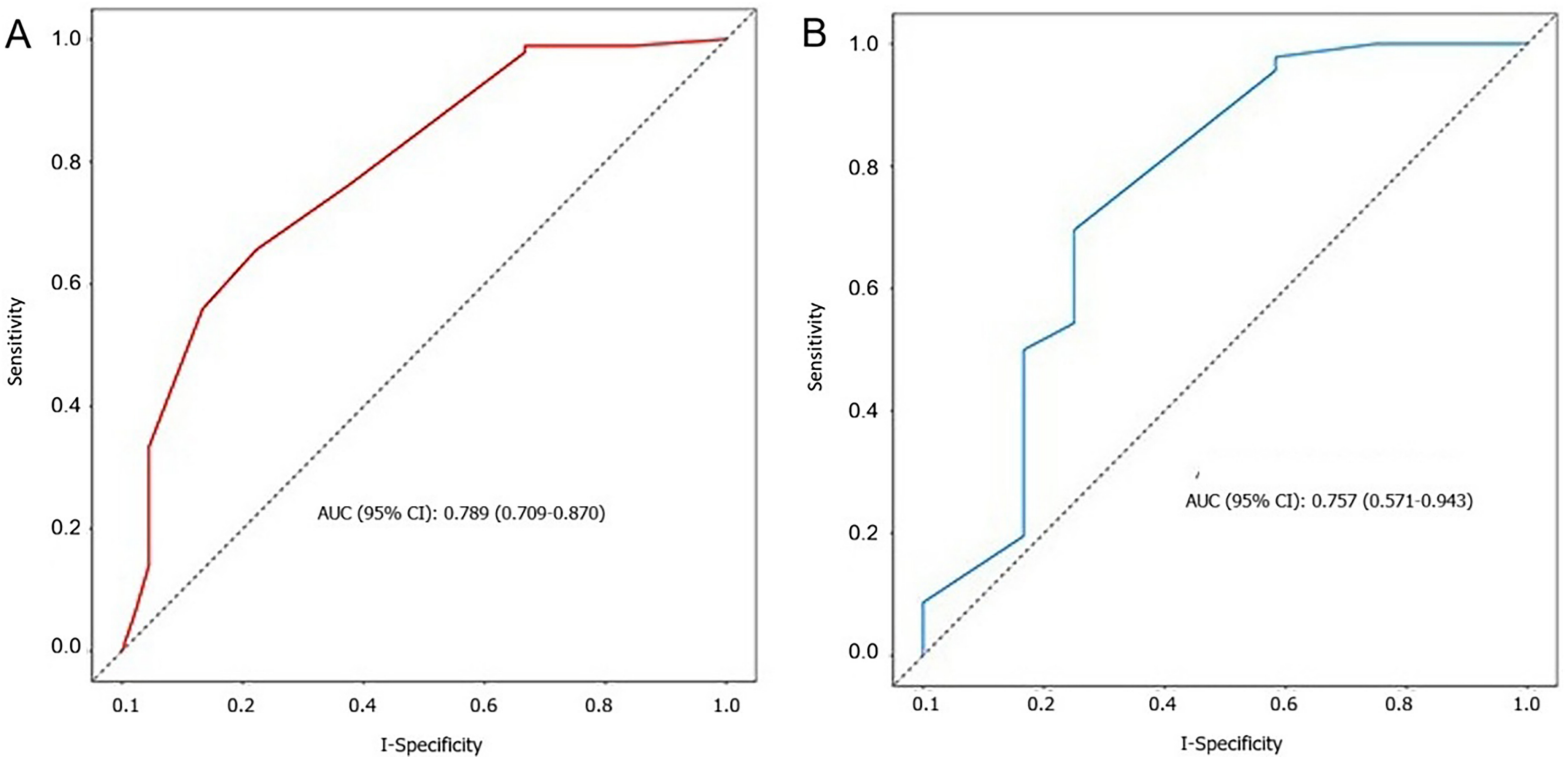
Receiver operating characteristic (ROC) curves of the predictive model. **(A)** ROC curve for the derivation cohort showing an area under the curve (AUC) of 0.789 (95% CI = 0.709–0.870). **(B)** ROC curve for the validation cohort showing an AUC of 0.757 (95% CI = 0.571–0.943), indicating acceptable discriminatory performance of the nomogram.

**FIGURE 4:**
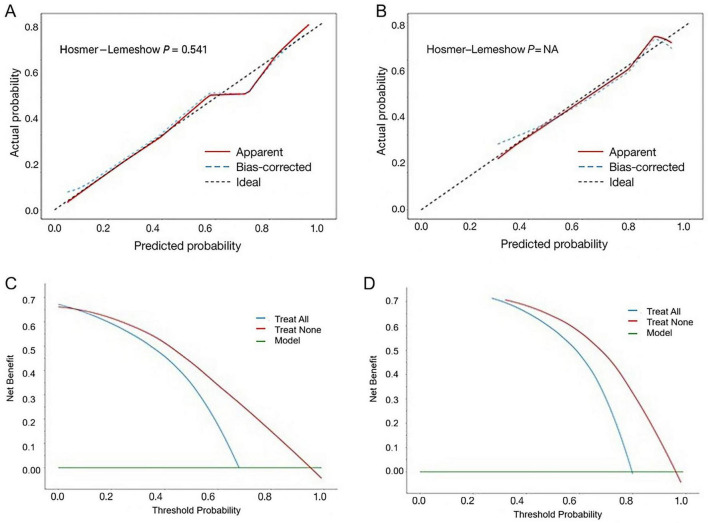
Calibration and decision curve analysis (DCA) of the nomogram. **(A)** Calibration curve for the derivation cohort demonstrating acceptable agreement between predicted and observed probabilities of s-DEACMP (Hosmer–Lemeshow goodness-of-fit test, *P* = 0.541). **(B)** Calibration curve for the validation cohort; due to the limited sample size, formal Hosmer–Lemeshow testing was not applicable, and calibration was assessed visually. **(C)** Decision curve analysis in the derivation cohort showing that the nomogram provides greater net clinical benefit than “treat-all” and “treat-none” strategies across a wide range of threshold probabilities. **(D)** Decision curve analysis in the validation cohort suggesting potential clinical utility of the predictive model.

Based on the four independent predictors identified, a nomogram was developed to estimate the individual risk of s-DEACMP (Figure 2). No significant differences were observed in the four final-model predictors between the derivation cohort (*n* = 140) and the validation cohort (*n* = 60), suggesting good comparability of the key model variables between the two groups (Table 4). The predictive model showed satisfactory discriminatory performance, with an area under the receiver operating characteristic curve (AUC) of 0.789 (95% CI = 0.709–0.870) in the derivation cohort and 0.757 (95% CI = 0.571–0.943) in the validation cohort (Figure 3). Calibration analysis demonstrated acceptable agreement between predicted and observed probabilities of s-DEACMP in both cohorts, supported by the Hosmer–Lemeshow goodness-of-fit test in the derivation cohort (χ^2^ = 5.023, *P* = 0.541) (Figures 4A,B). Furthermore, decision curve analysis (DCA) indicated that the nomogram provided greater net clinical benefit across a broad range of threshold probabilities compared with “treat-all” and “treat-none” strategies (Figures 4C,D). However, given the relatively small validation cohort and wide confidence interval of the validation AUC, the predictive performance of the model should be interpreted cautiously and requires confirmation in external multicenter cohorts. The nomogram based on four readily available clinical predictors demonstrated acceptable discrimination, calibration, and potential clinical utility for identifying patients at increased risk of s-DEACMP during acute hospitalization.

**TABLE 4:** Comparison of final-model predictors between the derivation and validation cohorts.

Variables	Derivation cohort (*n* = 140)	Validation cohort (*n* = 60)	*P*-value
Age > 40 years, n (%)			0.927
No	18 (12.86)	8 (13.33)	
Yes	122 (87.14)	52 (86.67)	
Disturbance of consciousness > 24 h, n (%)			0.350
No	83 (60.14)	39 (67.24)	
Yes	55 (39.86)	19 (32.76)	
Hypertension, n (%)			0.470
No	96 (68.57)	38 (63.33)	
Yes	44 (31.43)	22 (36.67)	
Hyperhomocysteinemia, n (%)			0.920
No	97 (69.29)	42 (70.00)	
Yes	43 (30.71)	18 (30.00)	

## Discussion

Severe delayed encephalopathy after acute carbon monoxide poisoning (s-DEACMP) represents one of the most debilitating complications of ACMP, yet its pathogenesis remains incompletely understood and reliable tools for risk stratification during acute hospitalization are lacking. Identifying clinically relevant risk predictors is therefore important for improving recognition of patients at increased risk for severe neurological impairment. In the present study, we systematically evaluated potential predictors of s-DEACMP and developed a preliminary risk stratification model based on readily available clinical and laboratory parameters. Our findings demonstrated that age > 40 years was independently associated with s-DEACMP. This observation is consistent with previous studies (3, 6–9) and may be explained by age-related declines in tolerance to hypoxia, progressive impairment of central nervous system resilience, and deterioration of cerebrovascular integrity and cerebral energy metabolism (10, 11). In addition, aging is associated with immune dysregulation and a higher prevalence of vascular comorbidities, such as arteriosclerosis and dyslipidemia, which may further amplify vulnerability to hypoxic–ischemic brain injury (12). However, the large odds ratio and wide confidence interval observed for age > 40 years should be interpreted cautiously, as the very high proportion of older patients in the s-DEACMP group may have introduced sparse-data bias and estimation instability. Therefore, although advanced age appears to be directionally associated with severe DEACMP, the precise magnitude of risk remains uncertain. Prolonged disturbance of consciousness (>24 h) was another independent predictor of s-DEACMP, highlighting the importance of neurological assessment during acute hospitalization. Sustained disturbance of consciousness likely reflects more severe initial cerebral hypoxia and ischemia, which can exacerbate white matter damage and promote irreversible demyelination. Advanced neuroimaging studies have shown that prolonged disturbance of consciousness is associated with restricted diffusion in cerebral white matter tracts, supporting its role as a marker of severe neuronal injury (13–15). We also identified hypertension as an independent risk factor for s-DEACMP. Chronic hypertension compromises cerebrovascular autoregulation and baseline cerebral perfusion, thereby reducing the brain’s capacity to withstand hypoxic stress. When combined with acute carbon monoxide–induced hypoxia, this impaired vascular reserve may accelerate demyelination and exacerbate neurological sequelae, offering a plausible explanation for the higher incidence of severe delayed encephalopathy in hypertensive patients.

Notably, this study identified hyperhomocysteinemia as a potentially relevant predictor associated with s-DEACMP. Although elevated homocysteine levels are well established as risk factors for cardiovascular and cerebrovascular diseases and for neurodegenerative disorders such as Alzheimer’s and Parkinson’s disease, their role in delayed encephalopathy after CO poisoning has received limited investigation. Our findings are consistent with those reported by Ren and Zhou (16), who demonstrated that elevated homocysteine levels were independently associated with cognitive dysfunction after acute carbon monoxide poisoning. Their results support the hypothesis that homocysteine-related vascular injury, oxidative stress, endothelial dysfunction, and neurotoxicity may contribute to adverse neurological outcomes following CO exposure, thereby lending further support to the potential relevance of hyperhomocysteinemia in patients at risk for severe DEACMP. Homocysteine exerts neurotoxic effects through multiple mechanisms, including endothelial dysfunction, microthrombus formation, oxidative stress, mitochondrial injury, and excitotoxic neuronal apoptosis mediated by N-methyl-D-aspartate receptor overactivation (17–19). Nevertheless, the association between hyperhomocysteinemia and s-DEACMP should be interpreted cautiously, particularly given the borderline statistical significance observed in baseline analyses and the limited existing clinical evidence specifically linking homocysteine to delayed encephalopathy after CO poisoning. Whether homocysteine-lowering interventions may influence neurological outcomes remains uncertain and requires prospective investigation.

Previous investigations into DEACMP have proposed a wide range of predictors, including inflammatory biomarkers (IL-6, MBP, S100B, NSE, GFAP), reduced neuroprotective factors (netrin-1), abnormal neuroelectrophysiological findings (SEP, VEP, BAEP), imaging abnormalities, and genetic polymorphisms (5). More recently, Kim et al. (20) introduced the COGAS scoring system to predict poor neurocognitive outcomes after ACMP. Unlike the COGAS model, which focuses on overall neurocognitive prognosis following ACMP, the present study specifically evaluated predictors associated with severe DEACMP defined by functional disability. In addition, the present model incorporated prolonged disturbance of consciousness observed during acute hospitalization, and was therefore intended for risk stratification prior to the onset of delayed encephalopathy rather than at the time of initial presentation. However, a validated model specifically targeting severe delayed encephalopathy has remained unavailable. In this context, our study contributes by developing a nomogram integrating four readily obtainable predictors—age >40 years, prolonged disturbance of consciousness, hypertension, and hyperhomocysteinemia. The model demonstrated acceptable discrimination in both the derivation and validation cohorts and showed satisfactory calibration and potential clinical utility. The discrimination performance observed in the present study (validation AUC = 0.757) was lower than that reported in some recently published prediction models. For example, Wang et al. (21) developed a multicenter prediction model for delayed encephalopathy after carbon monoxide poisoning and reported AUC values of 0.944 in the training cohort, 0.849 in the internal validation cohort, and 0.872 in the external validation cohort. The superior performance of that model may be attributable to its substantially larger multicenter dataset, inclusion of additional predictive variables, and more complex modeling strategy. In contrast, the present nomogram was intentionally developed using a limited number of routinely available clinical and laboratory variables to enhance practicality and ease of application during acute hospitalization. However, given the retrospective single-center design, modest validation cohort size, and wide confidence interval of the validation AUC, the predictive performance of the model should be interpreted cautiously. By relying on routine clinical and laboratory data, this nomogram may assist in identifying patients at increased risk for s-DEACMP during acute hospitalization, although external validation is required before broader clinical application.

Several limitations of this study should be acknowledged. First, the retrospective design and single-center setting may introduce selection bias and limit the generalizability of the findings. In addition, the relatively high proportion of DEACMP and severe cases in this cohort may reflect referral bias related to the specialized hyperbaric oxygen treatment setting. Second, although the overall sample size was relatively large, the validation cohort was modest, and external multicenter validation is required to confirm the stability and reproducibility of the predictive model. Third, ADL assessment was performed at peak disease severity during hospitalization rather than at standardized follow-up time points, which may reflect transient maximal disability rather than long-term neurological outcome. Fourth, potentially informative predictors—such as detailed MRI lesion characteristics, serial carboxyhemoglobin measurements, inflammatory biomarkers, advanced neuroimaging diffusion metrics, genetic markers, and detailed hyperbaric oxygen therapy parameters—were unavailable or inconsistently documented in this retrospective cohort. Additionally, homocysteine status was determined using both acute laboratory measurements and documented prior medical history, which may have introduced heterogeneity in predictor definition. Finally, although hyperhomocysteinemia emerged as a potentially relevant predictor, causal relationships cannot be inferred from this observational study, and interventional studies are required to determine whether lowering homocysteine levels can effectively reduce the risk of s-DEACMP.

## Conclusion

Age > 40 years, prolonged disturbance of consciousness, hypertension, and hyperhomocysteinemia were associated with severe delayed encephalopathy after acute carbon monoxide poisoning in this retrospective cohort. A nomogram incorporating these predictors demonstrated acceptable predictive performance in internal validation and may assist in identifying patients at increased risk of s-DEACMP during acute hospitalization. However, external multicenter validation is required before broader clinical implementation. Future prospective studies are warranted to further validate this model and to explore the potential relationship between homocysteine metabolism and neurological outcomes following acute carbon monoxide poisoning.

## Data Availability

The original contributions presented in this study are included in this article/supplementary material, further inquiries can be directed to the corresponding author.
